# Molecular cloning, prokaryotic expression and induction characteristics of the sesquiterpene synthase gene (*AsSS15*) from the *Chi-Nan* germplasm (*Aquilaria sinensis*)

**DOI:** 10.1007/s12298-025-01640-z

**Published:** 2025-09-04

**Authors:** Zhenghan Bao, Peiwen Sun, Jianhe Wei, Xiaohong Fao, Feifei Lv, Yun Yang

**Affiliations:** 1 Key Laboratory of Bioactive Substances and Resources Utilization of Chinese Herbal Medicine, Ministry of Education & National Engineering Laboratory for Breeding of Endangered Medicinal Materials, Institute of Medicinal Plant Development, Chinese Academy of Medical Sciences and Peking Union Medical College, Beijing, 100193 China; 2 Hainan Provincial Key Laboratory of Resources Conservation and Development of Southern Medicine & Key Laboratory of State Administration of Traditional Chinese Medicine for Agarwood Sustainable Utilization, Hainan Branch of the Institute of Medicinal Plant Development, Chinese Academy of Medical Sciences and Peking Union Medical College, Haikou, 570311 China

**Keywords:** *Chi-Nan* germplasm, Sesquiterpene synthase, Gene cloning, Prokaryotic expression, Induction characteristics

## Abstract

Sesquiterpenoids are one of the secondary metabolites in agarwood which, is a profitable traditional Chinese medicine and spice. Agarwood is derived from the injured *Aquilaria sinensis*. *Chi-Nan* germplasm is a new chemotype of *A. sinensis* that exhibits a higher capacity of agarwood production and the diversity of sesquiterpenoid biosynthesis. To examine the mechanism of sesquiterpene generation from *Chi-Nan* germplasm*,* a 1812 bp-length *sesquiterpene synthase 15* (*AsSS15*) gene encoding 603 amino acids was obtained from the wounded branches. *AsSS15* showed significant expression levels in the stem and root that the major tissues of agarwood production, indicating that it was involved in agarwood formation. *AsSS15* expression was higher in wounded branches of *Chi-Nan* germplasms than that in ordinary germplasms of *A. sinensis*. The AsSS15 recombinant protein was successfully expressed, and catalyzed farnesyl pyrophosphate (FPP) substrates to generate nerolidol in vitro. These findings demonstrated that *AsSS15* significantly induces the expression of gene related to sesquiterpene biosynthesis in *Chi-Nan* germplasm. This study first elucidates potential mechanisms of sesquiterpene generation from *Chi-Nan* germplasms, which adds to the theory of sesquiterpene accumulation in *A. sinensis* and serves as a foundation for future research into the biosynthesis and application of *Chi-Nan* germplasm.

**Supplementary Information:**

The online version contains supplementary material available at 10.1007/s12298-025-01640-z.

## Instruction

*A. sinensis* is recognized as a rare and endangered medicinal plant classified within *Aquilaria* and *Gyrinops* (Family Thymelaeaceae), serving as the plant source for agarwood (Editorial Committee of Flora of China 2004). Agarwood is an aromatic resinous wood that serves as a widely utilized traditional Chinese medicine and spice. Agarwood can be generated from roots, branches, and stems subjected to biotic and abiotic stimuli (Mohamed and Rozi 2016). The China Pharmacopoeia (Chinese Pharmacopoeia Commission 2020) designates *A. sinensis* as the sole legitimate medicinal resource of agarwood in China, with growing tropical and subtropical regions (Yin et al. 2016). Agarwood has a wide variety of aromatic chemicals, in which sesquiterpenes and phenylethyl chromone are the main ingredients (Chen et al. 2012; Li et al. 2016).

Sesquiterpenoid is a significant plant secondary metabolite implicated in adaptive responses to external stressors (Yonekura-Sakakibara and Saito 2009). Sesquiterpenoid biosynthesis in plants primarily involves the mevalonic acid (MVA) and methylerythritol phosphate (MEP) pathways (Yu and Utsumi 2009). The acetyl-CoA and pyruvate/phosphoglyceraldehyde are the precursors that begin sesquiterpene production, which need a cascade of enzymes, culminating in the production of isopentenyl diphosphate (IPP). The IPP and dimethylallyl pyrophosphate (DMADP) are converted to farnesyl pyrophosphate (FPP) by IPP isomerase and isopentenyltransferase. (Newman and Chappell 1999; Christianson 2007; Liang et al. 2002). The sesquiterpene skeleton structure is subsequently generated by the sesquiterpene synthase. The sesquiterpene synthase is the rate-limiting enzyme in these pathways (Degenhardt et al. 2009).

Currently, multiple sesquiterpene synthase genes have been cloned from plant species. Transgenic plants with excessive expression of sesquiterpene synthase genes, such as grapes (Dueholm et al. 2019), maize (Saldivar et al. 2023), rice (Zang et al. 2011) and *Pityopsis ruthii* flowers (Chen et al. 2023), can synthesize and accumulate high levels of sesquiterpenes. Sesquiterpene synthases play a crucial role in sesquiterpene biosynthesis. The cloning and verification of a few sesquiterpene synthase genes relevant to agarwood sesquiterpenoid synthesis have been a critical section of the sesquiterpene biosynthetic pathway (Kumeta and Ito 2010; Xu et al. 2013; Ye et al. 2018).

*Chi-Nan* germplasm, as a new chemotype of *A. sinensis*, is cultivated in southern China and has a capacity to produce agarwood with high content of resin (Hou et al. 2022). The agarwood from *Chi-Nan* germplasm has richer sesquiterpoids and phenylethyl chromones than ordinary germplasm in *A. sinensis* (Hou et al. 2022; Yu et al. 2021). After drilling, comparative transcriptome analysis revealed that the expression levels of genes related to defense reaction and sesquiterpene biosynthesis differed obviously between *Chi-Nan* germplasm and ordinary *A. sinensis*, in which the expression levels of *AsSS15* were higher *Chi-Nan* germplasm than in ordinary germplasm (Lv et al. 2022).

This study investigated sesquiterpene biosynthesis in the *Chi-Nan* germplasm from *A. sinensis*. The complete coding sequence (CDS) of *AsSS15* was cloned from the *Chi-Nan* germplasm. Bioinformatic analyses were employed to characterize the deduced AsSS15 protein and predict its functional properties. Quantitative real-time PCR (qRT-PCR) was used to determine the spatial expression profile of *AsSS15* across various tissues and to monitor its transcriptional dynamics in response to wounding in branches. The AsSS15 protein was subsequently expressed in a prokaryotic system and purified. Its enzymatic function was confirmed through western blot analysis and in vitro catalytic assays, which demonstrated its specific activity in sesquiterpene synthesis. These findings contribute significantly to elucidating the molecular mechanisms underlying sesquiterpene production in the *Chi-Nan* germplasm and provide valuable genetic data for future variety identification and breeding programs aimed at enhancing sesquiterpene yield in *A. sinensis*.

## Materials and methods

### Plant materials

Three-year-old *Chi-Nan* germplasm and ordinary germplasm (authenticated by DNA barcoding as *A. sinensis* (Lour.) Spreng.) were planted in an experimental base at the Hainan Branch of the Institute of Medicinal Plant Development (Haikou, China; 20°01′N, 110°25′E). The healthy and well-grown branches (diameter = 1.0 ± 0.2 cm) were selected for wound induction. The branches were cut and longitudinally scratched on the outside. The injured stem sections treated were encased in the clear bag with a small pore. At 30 days post-wounding, the 2 cm segments from the apical wounded regions were excised, and bark was removed. Snap-frozen in liquid nitrogen and stored at - 80 °C for RNA extraction. Healthy stems as control samples were harvested immediately. Three biological replicates were processed independently.

### Molecular cloning of the full-length of AsSS15

Total RNA was isolated from wound-induced branches of the *Chi-Nan* germplasm using the EASYspin Plus Plant RNA Rapid Extraction Kit (Aidlab Biotech, China) following the manufacturer’s protocol. The RNA samples integrity was verified by 1.5% agarose gel electrophoresis, and concentrations were quantified by spectrophotometer (NanoDrop 2000, Thermo Fisher). First-strand cDNA was synthesized from 1 μg total RNA using the TransScript One-step gDNA Removal and cDNA Synthesis SuperMix (TransGen Biotech, China). The full-length coding sequence (CDS) of *AsSS15* was retrieved from the published transcriptome dataset (Lv et al. 2022). Gene-specific primers (Table 1) were designed with Primer Premier 5.0 to amplify the *AsSS15* CDS. PCR products were purified and ligated into the​pEASY-Blunt Simple Cloning Vector (TransGen Biotech, China). The recombinant plasmid was transformed into* E*.* coli* Trans1-T1 phage-resistant chemically competent cells (TransGen Biotech), and positive clones were screened by colony PCR. Sanger sequencing of single-colony isolates was performed (Guangzhou Aiji Biotechnology Co, China).

**Table 1:** Primers for gene cloning and qRT-PCR detection

Primer purpose	Oligo name	Sequences (5′ to 3′)
Full-length CDS cloning	*TPS15*-cloning	F: ATGTCTTGCTTCCAAGCTCTT R: ATAAGGGATTGGATCTACAAGTATG
*AsSS15* expression	*TPS15*-PCR	F: TGCTAAAGAAGAGGTGAAAAGGG R: CCGCAAGGTCGTCATAGAGC
Reference gene	GADPH	F: CTGGTATGGCATTCCGTGTA R:AACCACATCCTCTTCGGTGTA

### Bioinformatics analysis of *AsSS15*

The functional domains of the *AsSS15*-encoded protein were determined using InterProScan, with specific catalytic residues annotated based on conserved motif alignment. Physicochemical properties were predicted via ProtParam (http://www.expasy.ch/tools/protparam.html). Transmembrane domain was assessed by TMHMM 2.0. Homologous proteins were identified by BLASTP against the NCBI database, and a neighbor-joining (NJ) phylogenetic tree was reconstructed in MEGA5 using the Poisson correction model with 1000 bootstrap replicates to evaluate clade support. The secondary structure of the protein was predicted employing the SOPMA web server, and homology modeling of the tertiary structure was performed using the Swiss-Model online platform, generating 3D structural models based on template recognition and automated comparative modeling.

### Expression characteristics of *AsSS15*

Tissue-specific and wound-induced expression profiles of *AsSS15* were quantified by qRT-PCR using LightCycler^®^ 96 System (Roche Diagnostics, Switzerland). Reactions were performed in a 20 μL volume containing: 1 μL cDNA, 1 μL gene-specific primers (10 μM; *AsSS15-F/R*), 10 μL of 2 × SYBR Premix Ex Taq™ (TransGen Biotech, China), and 7 μL ddH_2_O. Three technical replicates per biological sample were analyzed. The thermal cycling protocol comprised: initial denaturation at 94 °C for 8 min; 40 cycles of denaturation at 95 °C for 10 s, annealing at 56 °C for 15 s, and extension at 72 °C for 20 s; followed by a melt curve (95 °C for 10 s, 65 for 60 s, 97 °C for 1 s). Glyceraldehyde-3-phosphate dehydrogenase (GAPDH) served as the reference gene with primer sequences listed in Table 1. Relative gene expression was calculated using the comparative ^ΔΔ^Ct method (Schmittgen and Livak 2008).

### Prokaryotic expression of *AsSS15*

The *AsSS15* coding sequence was optimized using the prokaryotic expression codon optimization software to enhance translational efficiency in *E. coli*. Eight rare codons were replaced with *E. coli*-preferred synonyms while preserving the native amino acid sequence. Restriction sites for NdeI (5′-CATATG-3′) and XbaI (5′-TCTAGA-3′) were incorporated at the 5′ and 3′ termini, respectively. The optimized gene was synthesized and cloned into pCzn1 expression vector (Nanjing Zhongding Biotechnology Co, China). After sequencing, the recombinant plasmid was transformed into the *E. coli* strain BL21(DE)3PlyssTM for induced expression.

Transformed colonies were inoculated in the LB medium supplemented with 50 μg/mL Amp and incubated at 37 °C with shaking (220 rpm) until OD600 reached 0.6–0.8. Protein expression was induced with 0.4 mM isopropyl β-D-1-thiogalactopyranoside (IPTG) for 4 h at 37 °C and shaken at 11 °C overnight, respectively, to enhance soluble folding. Cells were harvested by centrifugation and lysed via sonication. The mixture was centrifuged to separate soluble and insoluble fractions. Target protein expression was analyzed by 12% SDS-PAGE with Coomassie Brilliant Blue R-250 staining.

### The detection of recombinant protein

The recombinant AsSS15 protein was identified by western blotting with anti-His tag primary antibodies. Antibody-bound complexes were incubated with horseradish peroxidase (HRP)-conjugated goat anti-rabbit IgG secondary antibodies for 1 h at room temperature. Protein signals were visualized using the West Pico Enhanced Chemiluminescent (ECL) Substrate (Solarbio, China) and imaged on the ChemiDoc™ XRS + Imaging System (Bio-Rad, USA) with automatic exposure optimization.

### Enzymatic function assays of *AsSS15*

The enzymatic reaction was performed containing 25 mM Tris–HCl (pH 7.0), 10% (v/v) glycerol, 10 mM MgSO_4_, 5 mM dithiothreitol (DTT), 60 µM farnesyl pyrophosphate (FPP), and 40 µg/mL recombinant protein. The mixture was vortex-mixed and incubated at 30 °C for 2 h. Sesquiterpene products were analyzed by solid-phase microextraction (SPME) using an Agilent 7890A system (Agilent Technologies, USA) equipped with an HP-5MS 5% Phenyl Silox capillary column (30 m × 0.25 mm i.d., 0.25 μm film thickness).

The ionization mode was EI with 70 eV. The carrier gas was helium, and the flow rate was 1 mL/min. The inlet temperature was set to 250 °C, and the column temperature was originally held at 80 °C for 2 min; then, the temperature was increased to 220 °C at a rate of 5 °C/min and held for 5 min. Compounds were identified by matching mass spectra to the NIST 11 reference database (National Institute of Standards and Technology).

For quantitative analysis, a nerolidol standard curve was created by spiking the reaction system with 20 to 60 μM nerolidol (in 10 μM increments) while maintaining constant total volume. Specific enzyme activity (nmol min^−1^ mg^−1^) was calculated based on nerolidol yield derived from the standard curve.

## Results

### Cloning and bioinformatics analysis of *AsSS15* in *Chi-Nan* germplasm

**Fig. 1:**
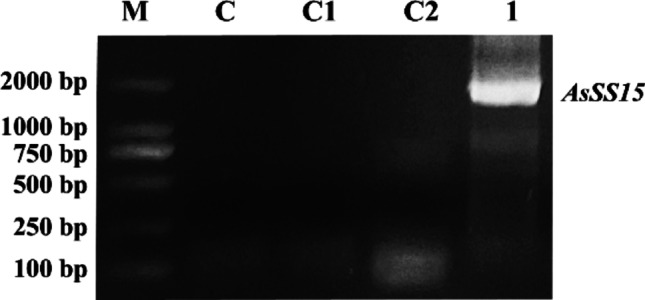
The PCR amplification of full-length Sequences of *AsSS15.* Lane M, DL2000 DNA marker. Lane C, C1 and C2, PCR control without DNA template. Lane 1, PCR amplification product of the full-length AsSS15 sequence

**Fig. 2:**
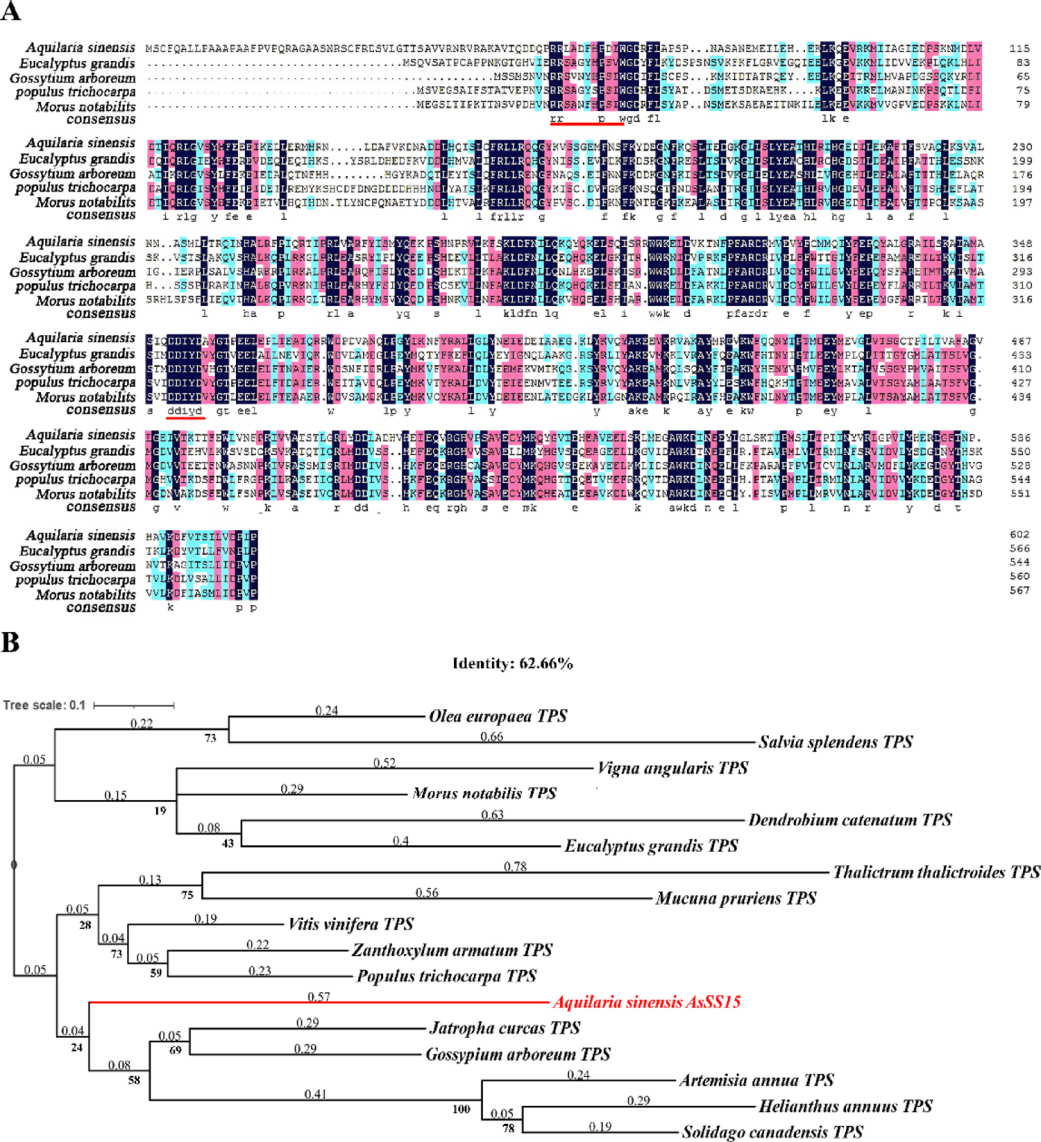
Aligment of amino acid sequences and phylogenetic tree of AsSS15. **A** Multiple alignment of amino acid sequences of AsSS15 protein in five plant species containing cloning sequence. Conserved sequences are depicted in dark blue, while differing residues are illustrated in pink and blue. The Red underlines indicate TPS preserved motifs Rx8W and DDxxD. **B** The NJ phylogenetic tree based on the amino acid sequence of AsSS15 and other homologues sequences

A DNA sequence was obtained in wounded branches of *Chi-Nan* germplasm by PCR amplification. The amplification product exhibited a clear and bright single band in agarose gel electrophoresis, compared with the no-template control (NTC) lane in which no detectable amplification (Fig. 1). The size of the amplified fragment corresponded to the sequence derived from transcriptomic data. After sequencing, it was found that the size of obtained sequence was 1812 bp. Bioinformatic analysis revealed a complete open reading frame (ORF) encoding a 603-amino acid protein. Conserved domain prediction using the NCBI database identified signature motifs characteristic of sesquiterpene synthases: the R(R)X_8_W motif (residues 59 ~ 69) and DDxxD motif (residues 352 ~ 356). Sequence alignment demonstrated more than 62.66% identity with geranyl synthases from *Eucalyptus grandis*, *Gossypium arboreum*, *Populus trichocarpa*, and *Morus notabilis* (Fig. 2A), confirming it was a sesquiterpene synthase gene. It was named *AsSS15* and the gene information was deposited in GenBank under accession number PQ586424.

A rooted neighbor-joining phylogenetic tree was constructed using the aligned amino acid sequences of AsSS15 and homologs from diverse plant species. The tree topology indicated that AsSS15 clustered closely with sesquiterpene synthases from *Artemisia annua*, *Helianthus annuus*, *Solidago canadensis*, *Gossypium arboreum*, and *Jatropha curcas* (Fig. 2B), suggesting strong functional conservation due to shared structural features.

Furthermore, it was predicted that AsSS15 is a hydrophilic protein with a molecular formula of C_3118_H_4858_N_834_O_903_S_24_, a molecular weight of 69.16 kDa, and a theoretical isoelectric point (pI) of 5.92. Secondary structure prediction indicated that α-helices constitute the predominant conformation (350 residues, 58.0% of total), followed by β-strands (22 residues, 3.7%), with the remainder comprising turns and random coils.

**Fig. 3:**
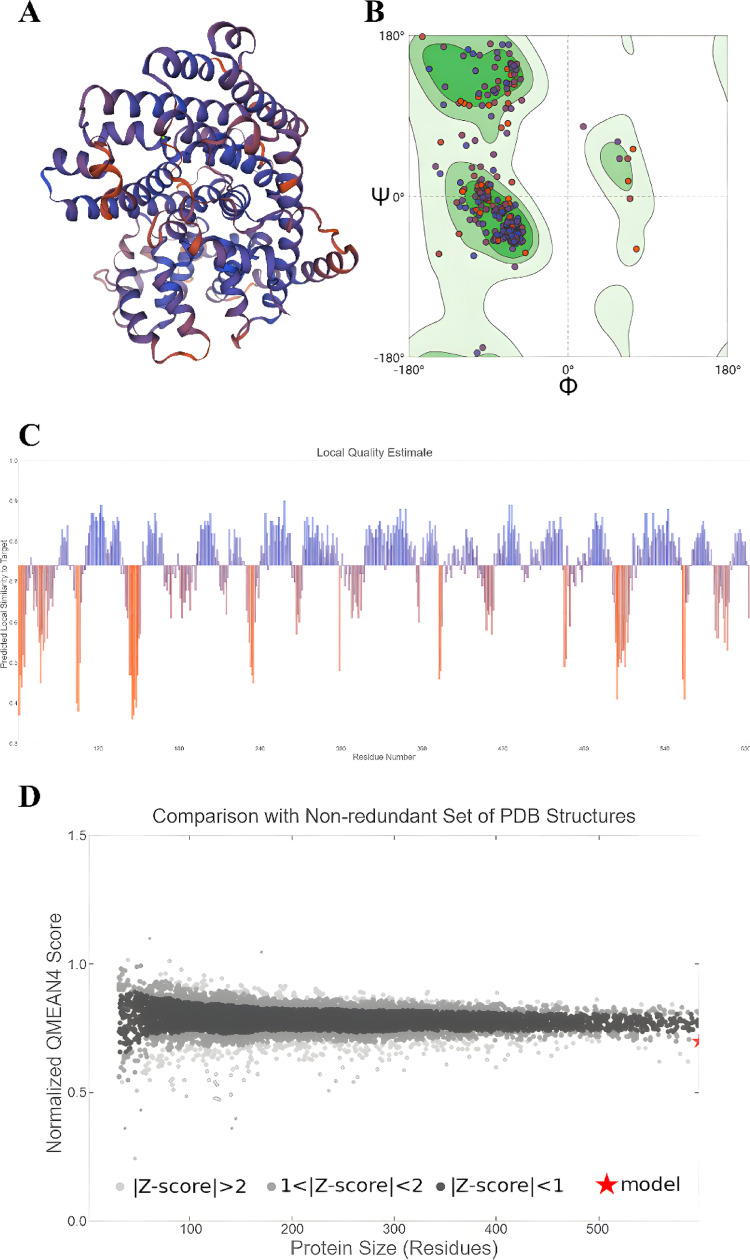
AsSS15 protein 3-D mode was predicted and analyzed. The tertiary structure of AsSS15 was created. **B** Ramachandran Plots analysis of AsSS15 3-D mode. **C** AsSS15 3-D mode quality estimation. **D** AsSS15 tertiary structure was compared with non-redundant Set of PDB Structures

The tertiary structure was modeled using SWISS-MODEL with 5-epi-aristolochene synthase (PDB: 4rnq.1.A) as a template. The model exhibited 42.19% sequence identity to the template and a QMEANDisCo Global score of 0.74 ± 0.05 (Fig. 3). Subcellular localization analysis predicted chloroplastic localization without transmembrane domains or signal peptides, consistent with a non-secretory intracellular protein.

### Expression characteristics of *AsSS15*

**Fig. 4:**
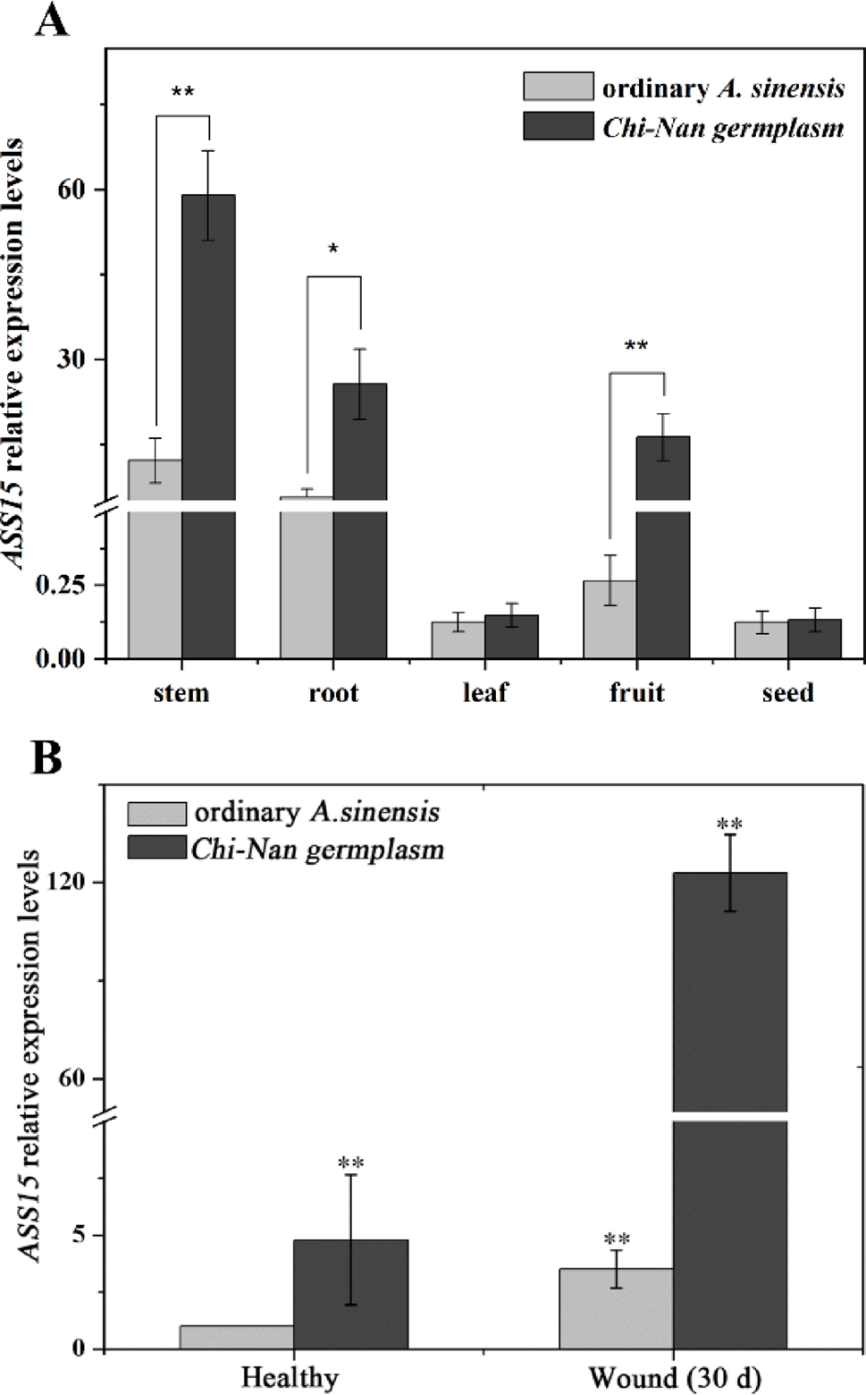
The gene expression characteristic analysis of *AsSS15.* **A **The relative expression levels of *AsSS15* in roots, stems, leaves, fruits and seeds of *Chi-Nan* germplasm and ordinary *A. sinensis*. **B** The wound-induced expression levels comparative of *AsSS15* in *Chi-Nan* germplasm and ordinary *A. sinensis.* (*, *p* ≤ 0.05; **, *p* ≤ 0.01)

To elucidate the function of *AsSS15*, its transcript abundance was quantified by qRT-PCR across multiple tissues (stems, roots, leaves, fruits, and seeds) of 3-year-old *Chi-Nan* germplasm. *AsSS15* was expressed in all tissues, with the highest levels in stems, followed by roots and fruits, while leaves and seeds showed the lowest expression (Fig. 4A). It was suggested that *AsSS15* played a potential role in agarwood biosynthesis. Comparative analysis revealed that *AsSS15* expression levels in healthy stems of *Chi-Nan* germplasm were significantly higher than in ordinary germplasm of *A. sinensis*. After being wounded, biological regulation of *ASS15* was markedly enhanced in *Chi-Nan* germplasm stems relative to ordinary *A. sinensis* (Fig. 4B). These results were indicated that wound-responsive *AsSS15* gene expression contributed to agarwood formation in *A. sinensis*. particularly in the *Chi-Nan* germplasm where expression was generally higher compared to ordinary germplasm of *A. sinensis*.

### Expression and purification of AsSS15 protein

**Fig. 5:**
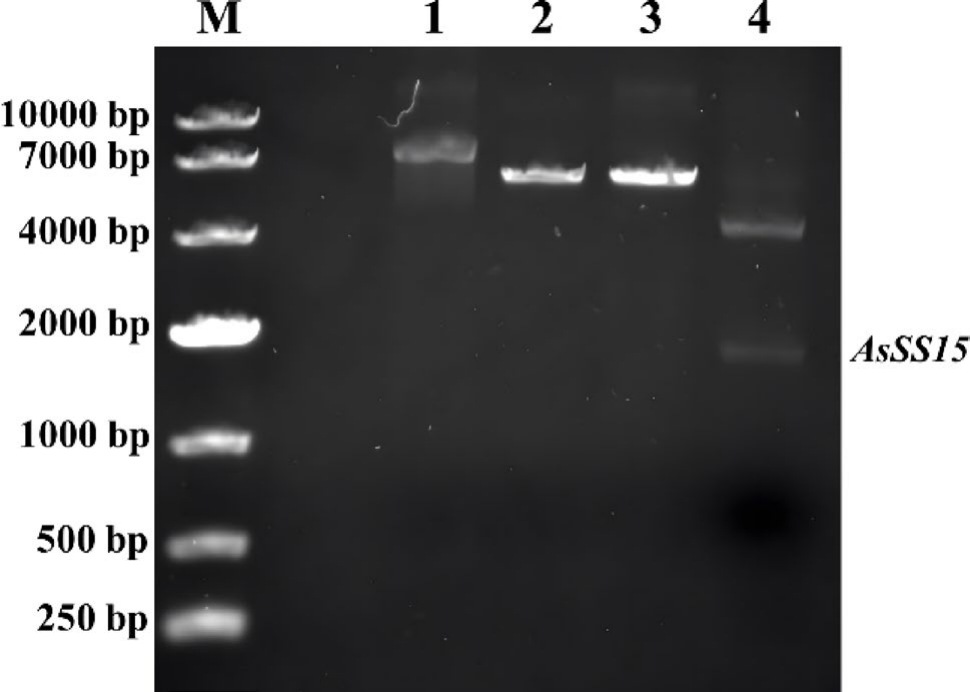
The double digestion of AsSS15-pCzn1 recombinant vector. Lane M, DL10000 DNA marker. Lane 1, AsSS15-pCzn1 with no-restriction enzyme digestion. Lane 2, AsSS15-pCzn1 with NedI digestion. Lane 3, AsSS15-pCzn1 with XbaI digestion. Lane 4, AsSS15-pCzn1 with double-restriction enzyme digestion

The *AsSS15* sequence was ligated into the expression vector pCzn1 to generate the recombinant plasmid AsSS15-pCzn1. This construct was subsequently transformed into BL21(DE3)Plyss™ competent cells. The recombinant plasmid was further validated through double-digestion with restriction enzymes, yielding two fragments whose sizes corresponded to the linearized vector (~ 4400 bp) and the AsSS15 insert (~ 1812 bp) (Fig. 5). These results confirmed the successful insertion of the *AsSS15* into the pCzn1.

**Fig. 6:**
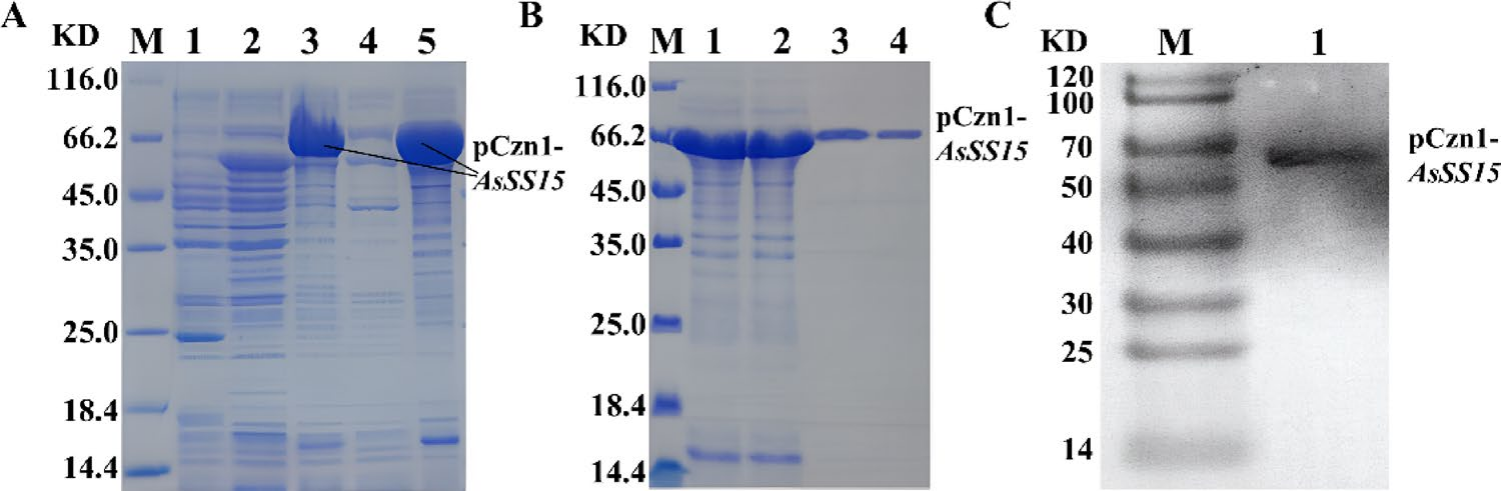
AsSS15-pCzn1 prokaryotic expression and detection. **A** AsSS15-pCzn1 protein was induced to express in E. coil BL21 (DE3) by SDS-PAGE. Lane M, protein maker (kD); Lane 1, pCzn1 with 0.4 mM IPTG induced for 4 h; Lane 2, AsSS15-pCzn1 without induction; Lane 3, AsSS15-pCzn1 with 0.4 mM IPTG induced for 4 h; Lane 4, supernatant for AsSS15-pCzn1 after fragmentation; Lane 5, sediment for AsSS15-pCzn1 after fragmentation. **B** Purification of AsSS15-pCzn1. Lane M, protein maker (kD). Lane 1, sediment of induced sample after fragmentation; Lane 2, AsSS15-pCzn1 in effluent; Lanes 3–4, recombinant protein in elution buffer. **C**. Western blot analysis of AsSS15-pCzn1. Lane M, protein molecular mass maker (kD); Lane 1, total protein of AsSS15-pCzn1 with 0.4 mM IPTG induced

The AsSS15-pCzn1 in *E. coli* strain BL21(DE)3PlyssTM was induced to express by IPTG. A prominent protein band of 66.2 kDa was generated (Fig. 6A), corresponding to the theoretical molecular weight of AsSS15-pCzn1. The recombinant protein predominantly accumulated in the insoluble fraction as inclusion bodies, consistent with typical expression patterns for plant-derived sesquiterpene synthases in bacterial systems. During denaturation, renaturation, and purification, the pure AsSS15-pCzn1 protein was obtained (Fig. 6B). Futuremore, a monospecific signal at 66.2 kDa was detected by Western blot with anti-His antibodies (Fig. 6C). This demonstrated that AsSS15-pCzn1 recombinant plasmid was successfully prokaryotic expressed.

### AsSS15 was involved in sesquiterpene biosynthesis

To validate the catalytic function of AsSS15, the recombinant protein was incubated with farnesyl diphosphate (FPP; C15substrate). Reaction products were analyzed by gas chromatography-mass spectrometry (GC–MS). A standard curve was established using nerolidol standards (20 ~ 60 μM). The linear regression equation (y = [slope]x + [intercept]) and correlation coefficient are summarized in Table 2, where x represents nerolidol concentration (μM) and y denotes peak area integrated from total ion chromatograms (TIC).

**Table 2:** The linear equation and correlation coefficient of AsSS15 catalytic efficienc

Method	Linear range/ μmol/L	Linear equation	R^2^
SPME-*AsSS15*	20–60	y = 2.19x–11.05	0.8867

**Fig. 7:**
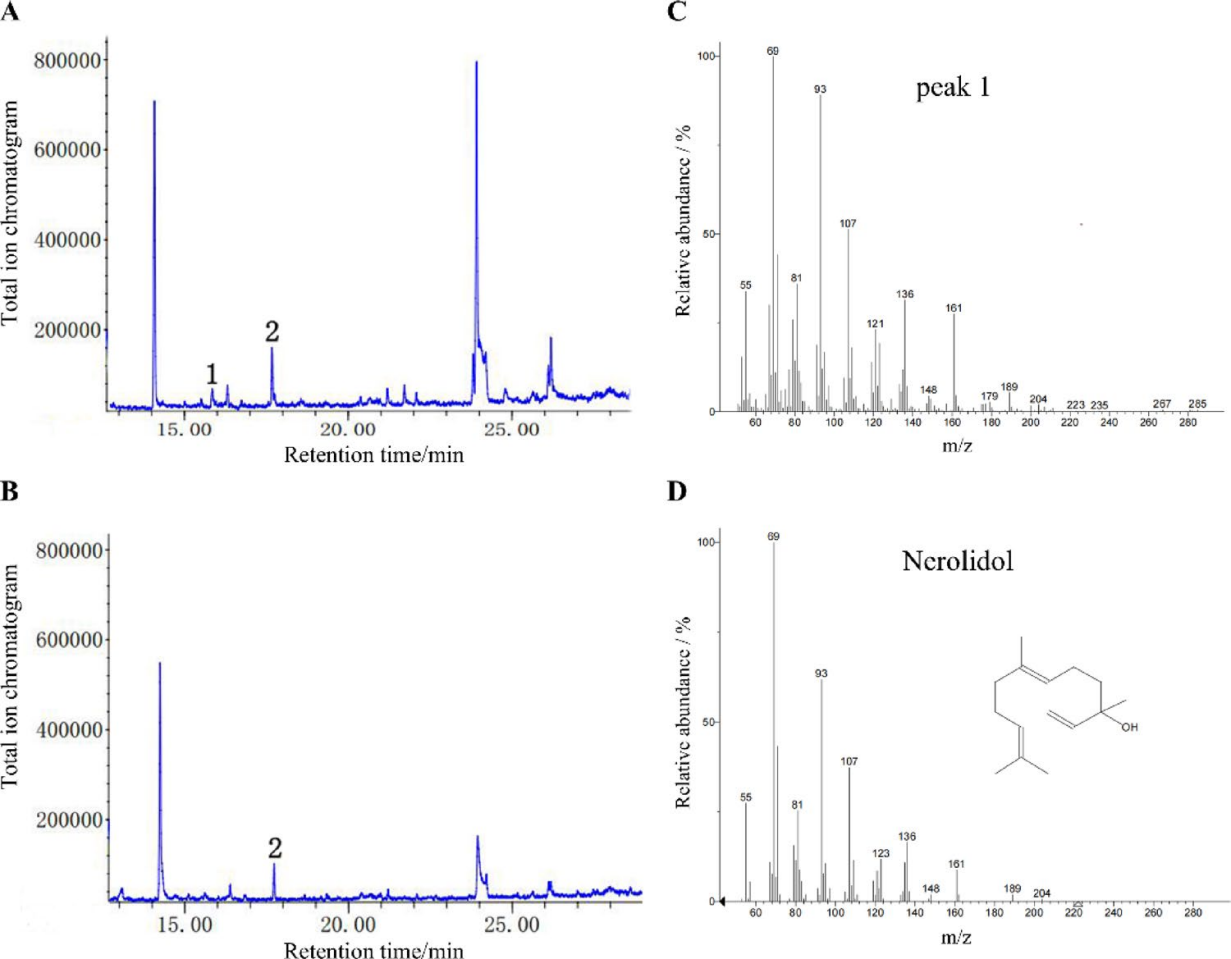
Total ion chromatogram of the products by AsSS15 catalysis. **A** Chromatogram of products catalyzed by AsSS15. **B** Chromatogram of blank sample. **C** the ion diagram of peak 1. **D** ion diagram of nerolidol in NIST database

GC–MS analysis revealed a distinct peak at 15.907 min in AsSS15-catalyzed reactions (Fig. 7A, B), which was absent in negative controls (enzyme omitted). Mass fragmentation patterns matched the NIST library reference for nerolidol (Fig. 7C, D), with diagnostic ions at m/z 69, 93, and 161. Based on the standard curve, the catalytic production of 14.36 μM nerolidol by the AsSS15 was calculated, corresponding to total activity of 0.1596 U/mL and the specific activity of the enzyme was 3.989 U/mg in the assay system. These results confirm that AsSS15 was a sesquiterpene synthase that could catalyze FPP to nerolidol.

## Discussion

Sesquiterpenoids represent essential secondary metabolite that confer resistance against biotic and abiotic stresses in plants (Habash et al. 2020; Liu et al. 2020). The biosynthesis of these compounds is catalyzed predominantly by sesquiterpene synthases (TPSs), which are critical for terpenoid profiles in plant (Li et al. 2021). Historically, molecular cloning and functional characterization of TPS genes have been pursued in crops, fruit trees, and medicinal species, aiming to exploit their anti-inflammatory/antimicrobial activities (Yu et al. 2023). *A. sinensis* served as the primary plant source of agarwood, which is a resinous wood formed after mechanical wounding or fungal infection (Ma et al. 2023). Recent studies identified the *Chi-Nan* germplasm as exhibiting accelerated agarwood formation and producing diverse sesquiterpenoid derivatives compared to ordinary germplasm *A. sinensis* (Yu et al. 2021). Consequently, cloning and functional analysis of *TPS* genes in *Chi-Nan* germplasm are pivotal for elucidating the molecular mechanisms underlying rapid agarwood formation.

The terpene synthase (*TPS*) gene family is generally considered a central hub governing terpenoid structural diversity (Karunanithi et al. 2019; Boncan et al. 2020). The *Chi-Nan* germplasm exhibits significantly enriched terpenoid profiles under mechanical injury, compared with ordinary *A. sinensis.* Previously, *AsSS15* was identified as markedly upregulated in wound-induced *Chi-Nan* germplasm by transcriptomic analyses. In this study, *AsSS15* was cloned and functionally identified to explore the rich sesquiterpene biosynthesis in *Chi-Nan* germplasm.

Structural prediction revealed that *AsSS15* contains conserved TPS catalytic motifs: an N-terminal RRX_8_W domain (59 ~ 69) and a C-terminal DDxxD motif (352 ~ 356), consistent with sesquiterpene synthase activity. Phylogenetic reconstruction demonstrated *AsSS15* was predominantly expressed in dicotyledonous plants, reflecting evolutionary trajectories driven by functional specialization. Dicot TPS genes are predominantly associated with ecological adaptation via secondary metabolism, where lineage-specific expansions amplify terpenoid diversity in response to biotic/abiotic stressors (Yan et al. 2023). Homology alignment confirmed significant sequence conservation between AsSS15 and sesquiterpene synthases from *Eucalyptus camaldulensis*, *Gossypium arboreum*, *Populus trichocarpa*, and *Morus notabilis*. It has been elucidated that TPS genes (such as *EcTPS*, *GaTPS*, and *PtTPS*) are positively influenced by both biotic and abiotic stressors, including reactive oxygen species, flooding, and insect herbivory, which can enhance the resistance of plants against environmental stressors (Cui et al. 2023; Huang et al. 2018; Irmisch et al. 2014; Kanagendran et al. 2018).

TPS genes have been expressed ubiquitously across a range of plant tissues, yet their tissue-specific expression patterns are distinct, leading to the synthesis of a broad array of secondary metabolites to assume varied activities. For example, α-pinene, the most profuse volatile terpenoid released by the flowers of Pityopsis ruthii, is not detectable in leaves, stems, and roots. (Chen et al. 2023). This spatial regulation enables plants to deploy terpenoids for ecological functions (e.g., pollinator attraction in flowers, herbivore deterrence in leaves) while minimizing metabolic costs (Xu et al. 2019). In this study, tissue-specific gene expression showed *AsSS15* transcription in roots, stems, leaves, fruits, and seeds of healthy trees. The highest expression levels were found in stems (peak), moderate levels were in roots and fruits, and minimal activity was in leaves and seeds, which was aligned with agarwood deposition patterns in wounded xylem. Crucially, mechanical stem injury triggered near 100-fold expression of *AsSS15*, confirming its pivotal role in wound-inducted sesquiterpenoid biosynthesis during agarwood formation.

Heterologous expression of eukaryotic genes in prokaryotic systems is frequently hampered by codon usage bias, leading to substantially reduced protein yields or translational failure (Sørensen et al. 1989; Zhang et al. 1991). Despite this constraint, in vitro enzymatic assays confirmed that recombinant AsSS15 protein catalytically converted farnesyl diphosphate (FPP) to nerolidol, that is a biologically active sesquiterpene alcohol with multifaceted pharmacological properties (Fonsêca et al. 2016; Iqubal et al. 2019). It was demonstrated that AsSS15 was a functional protein and was involved in the biosynthesis of sesquiterpenes.

In light of the greater variety of sesquiterpene synthase genes and the diverse array of sesquiterpene fractions facilitating the synthesis in *Chi-Nan* germplasm, it is imperative to conduct comprehensive investigations into the interactions between *AsSS15* and other sesquiterpene synthase genes, as well as its regulatory functions in the context of agarwood formation. Elucidating these mechanisms will establish novel molecular strategies to enhance agarwood quality and expand the utility of *A. sinensis* germplasm resources.

## Supplementary Information

Below is the link to the electronic supplementary material.

Supplementary Material 1

Supplementary Material 2

Supplementary Material 3

Supplementary Material 4

Supplementary Material 5
