# Supplementary figures and images for: Molecular cloning, prokaryotic expression and induction characteristics of the sesquiterpene synthase gene (AsSS15) from the Chi-Nan germplasm (Aquilaria sinensis)
Source: Physiol Mol Biol Plants. 2025 Sep 4;31(9):1421–31. doi: 10.1007/s12298-025-01640-z (PMC12534665; doi:10.1007/s12298-025-01640-z)

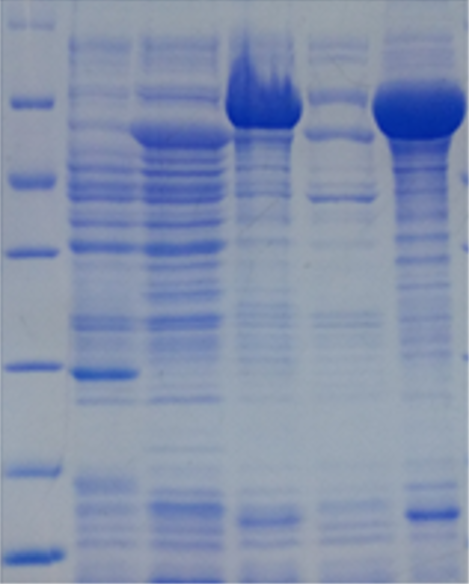

Supplement: Supplementary file 1 — Supplementary Material 1 [file 12298_2025_1640_MOESM1_ESM.tif]

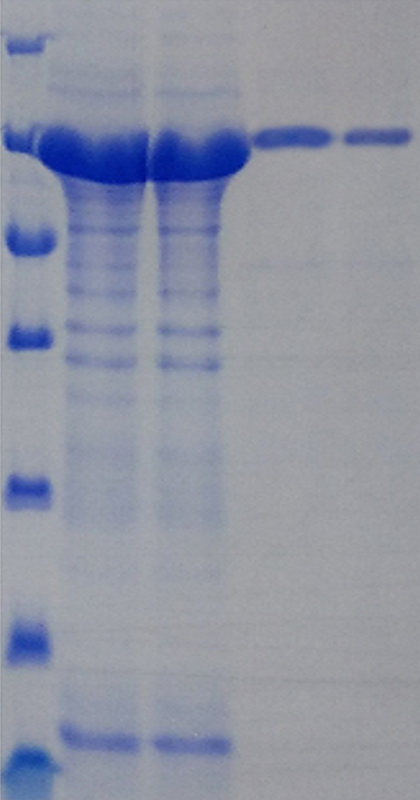

Supplement: Supplementary file 2 — Supplementary Material 2 [file 12298_2025_1640_MOESM2_ESM.tif]

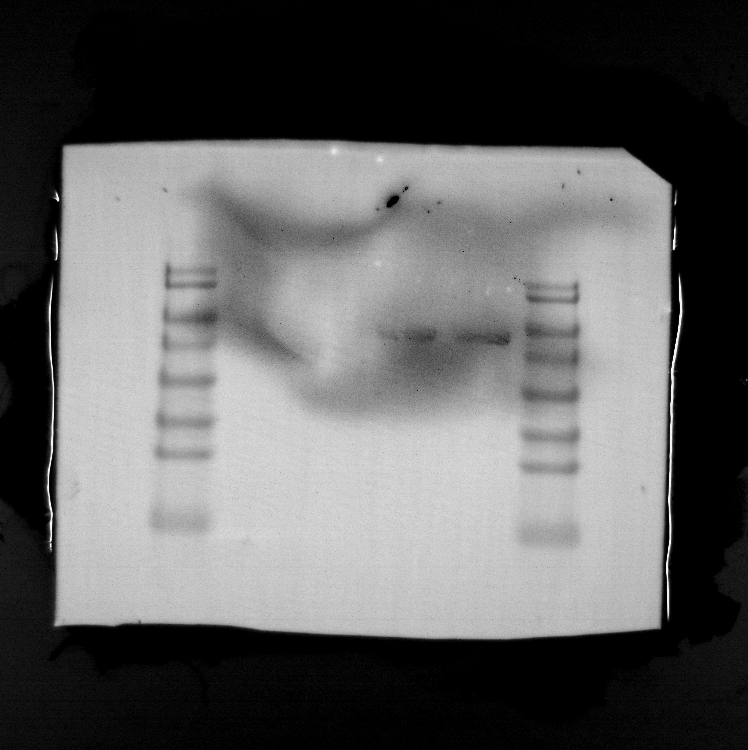

Supplement: Supplementary file 3 — Supplementary Material 3 [file 12298_2025_1640_MOESM3_ESM.tif]

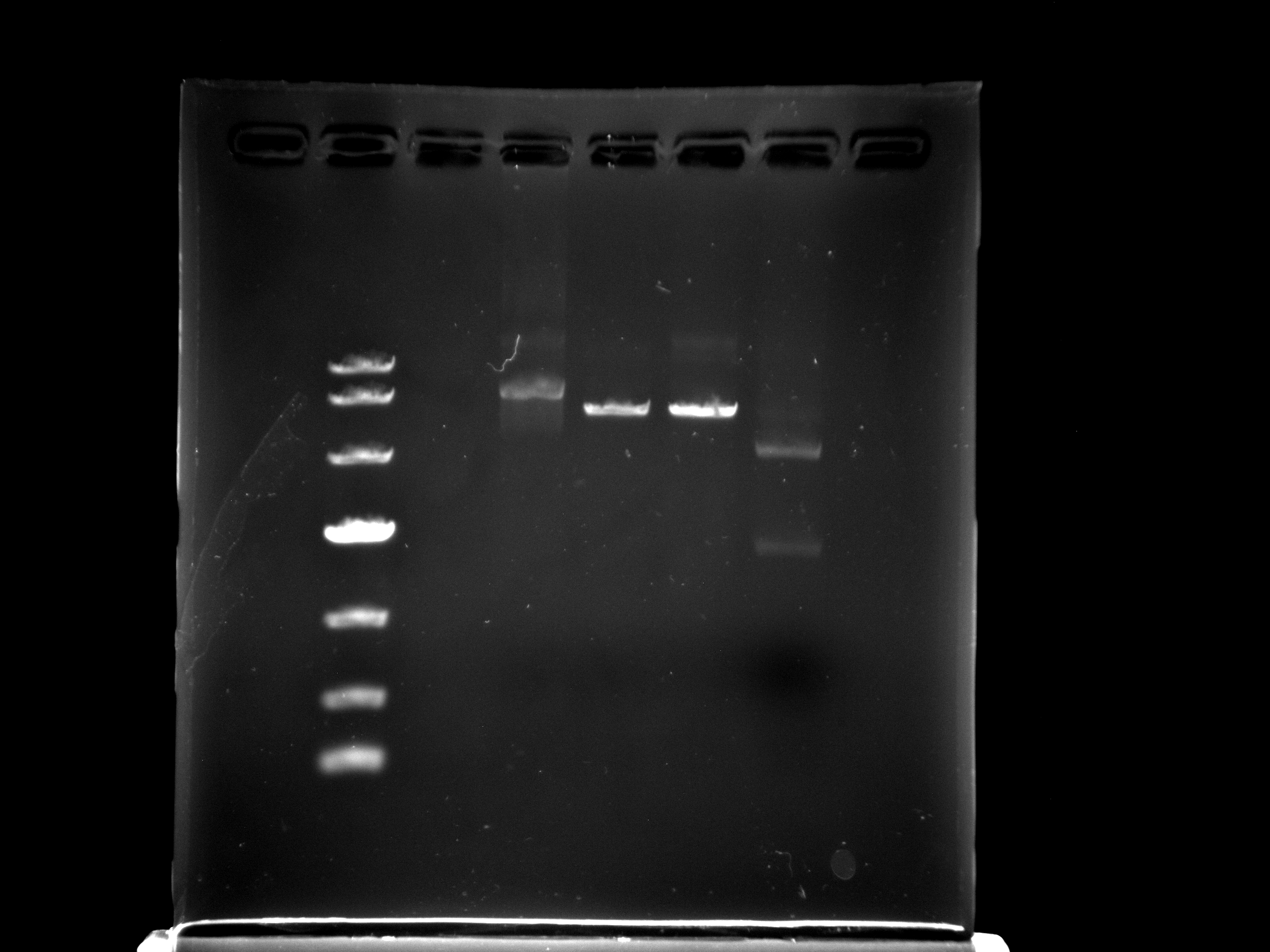

Supplement: Supplementary file 5 — Supplementary Material 5 [file 12298_2025_1640_MOESM5_ESM.tif]
